# Correction: CD90 and CD24 Co-Expression Is Associated with Pancreatic Intraepithelial Neoplasias

**DOI:** 10.1371/journal.pone.0176804

**Published:** 2017-04-25

**Authors:** The PLOS ONE Staff

## Notice of republication

This article was republished on March 15, 2017, to correct errors that were introduced during the typesetting process. The publisher apologizes for the error. Please download this article again to view the correct version. The originally published, uncorrected article and the republished, corrected article are provided here for reference.

## Supporting information

S1 File Originally published, uncorrected article. (PDF)

S2 File Republished, corrected article. (PDF)
