# Supplementary figures and images for: CD90 and CD24 Co-Expression Is Associated with Pancreatic Intraepithelial Neoplasias
Source: PLoS One. 2016 Jun 22;11(6):e0158021. doi: 10.1371/journal.pone.0158021 (PMC4917090; doi:10.1371/journal.pone.0158021)

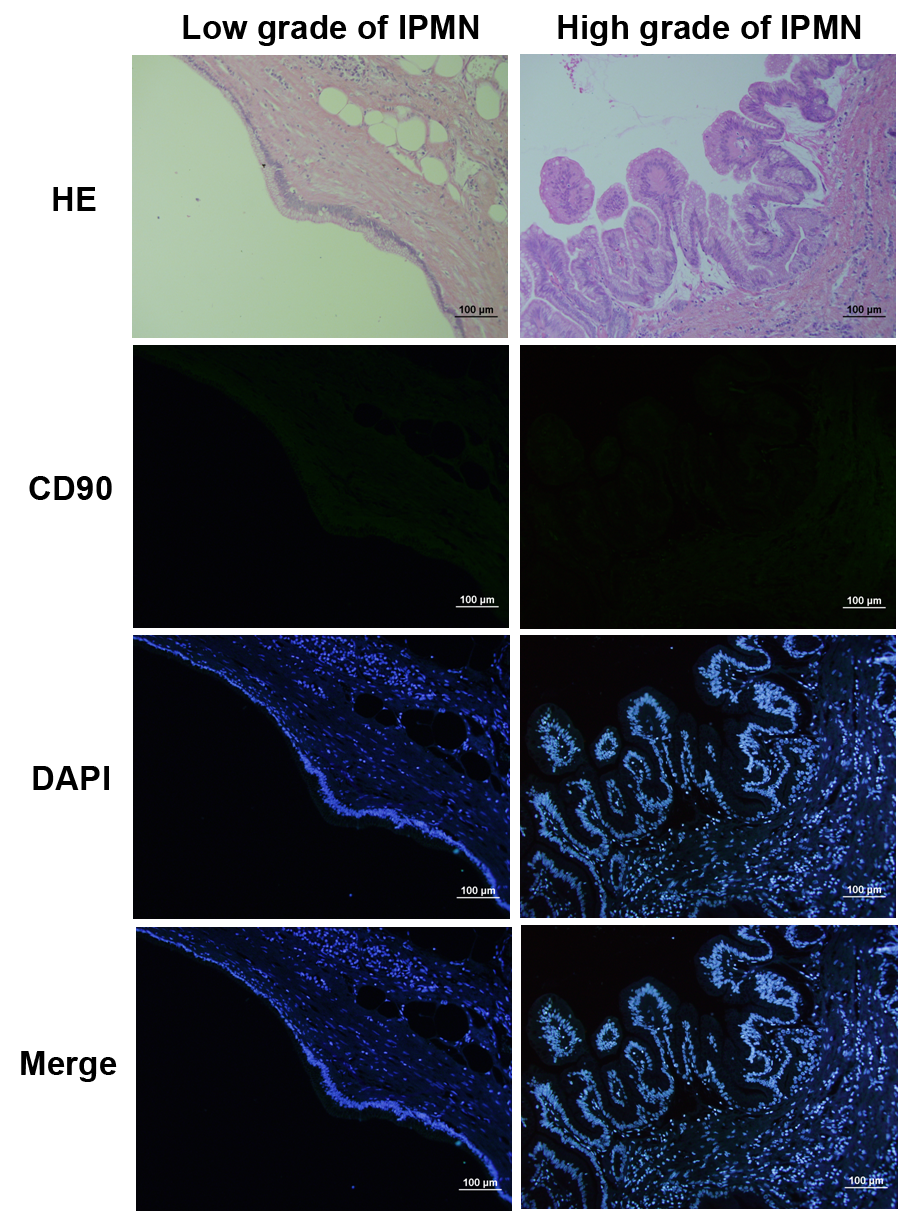

Supplement: S1 Fig — Nuclei visualization was shown by DAPI (blue) staining. There was no CD90 expression observed in stroma around the lesion ducts both in low and high grades of IPMN. Scale bars = 100 μm. (TIF) [file pone.0158021.s001.tif]
