# Supplementary material for: Correction: CD90 and CD24 Co-Expression Is Associated with Pancreatic Intraepithelial Neoplasias
Source: PLoS One. 2017 Apr 25;12(4):e0176804. doi: 10.1371/journal.pone.0176804 (PMC5404862; doi:10.1371/journal.pone.0176804)
Supplement: S1 File — (PDF) [file pone.0176804.s001.PDF]

RESEARCH ARTICLE

CD90 and CD24 Co-Expression Is Associated with Pancreatic Intraepithelial Neoplasias

Xiucong Pei[1,2], Jianhui Zhu[1], Rui Yang[1], Zhijing Tan[1], Mingrui An[1], Jiaqi Shi[3], David M. Lubman[1]*

1 Department of Surgery, University of Michigan Medical Center, Ann Arbor, Michigan, 48109, United States of America, **2** Department of Toxicology, School of Public Health, Shenyang Medical College, Liaoning, 110034, China, **3** Department of Pathology, University of Michigan School of Medicine, Ann Arbor, Michigan, 48109, United States of America

* dmlubman@umich.edu

CrossMark
click for updates

OPEN ACCESS

Citation: Pei X, Zhu J, Yang R, Tan Z, An M, Shi J, et al. (2016) CD90 and CD24 Co-Expression Is Associated with Pancreatic Intraepithelial Neoplasias. PLoS ONE 11(6): e0158021. doi:10.1371/journal.pone.0158021

Editor: Pankaj K Singh, University of Nebraska Medical Center, UNITED STATES

Received: March 24, 2016

Accepted: June 8, 2016

Published: June 22, 2016

Copyright: © 2016 Pei et al. This is an open access article distributed under the terms of the Creative Commons Attribution License, which permits unrestricted use, distribution, and reproduction in any medium, provided the original author and source are credited.

Data Availability Statement: All relevant data are within the paper and its Supporting Information files.

Funding: This work received support from three funding sources, which are listed below: (1) Grant R01. GM 49500 is supported by the U.S. National Institutes of Health; (2) Grant 81373029 is supported by the National Natural Science Foundation of China in China; (3) China Scholarship Council (no number) has supported the first author, Xiucong Pei, to work in the United States as a visiting scholar. The funders had no role in study design, data collection and analysis, decision to publish, or preparation of the manuscript.

Abstract

Thy-1 (CD90) has been shown to be a potential marker for several different types of cancer. However, reports on CD90 expression in pancreatic intraepithelial neoplasia (PanIN) lesions are still limited where PanINs are the most important precursor lesion of pancreatic ductal adenocarcinoma (PDAC). Herein, we investigate candidate markers for PanIN lesions by examining the distribution and trend of CD90 and CD24 expression as well as their co-expression in various stages of PanINs. Thirty cases of PanINs, which were confirmed histopathologically and clinically, were used to evaluate protein expression of CD90 and CD24 by immunofluoresence double staining. CD90 was found to be mainly expressed in stroma around lesion ducts while not observed in acini and islets in PanINs. CD90 also showed increased expression in PanIN III compared to PanIN III. CD24 was mainly present in the cytoplasm and membrane of pancreatic ductal epithelia, especially in the apical epithelium of the duct. CD24 had higher expression in PanIN III compared with PanIN IIIIII or PanIN III. CD90 was expressed around CD24 sites, but there was little overlap between cells that expressed each of these proteins. A correlation analysis showed that these two proteins have a moderate relationship with PanIN stages respectively. These results suggest that co-expression of CD90 and CD24 may have an important role in the development and progression of PanINs, which is also conducive to early detection and treatment of PDAC.

Introduction

Pancreatic intraepithelial neoplasia (PanIN) is known as the most important precursor of pancreatic ductal adenocarcinoma (PDAC), where PDAC has an especially high mortality rate. There is currently no specific or sensitive diagnostic method for early detection of this disease. Reports have shown that PanINs form gradually before PDAC through three grades PanIN III, PanIN IIIIII and PanIN III and evolve from a noninvasive lesion to invasive cancer [1,2]. Mutation in several cancer related genes including K-ras and p53 are known to be involved in

Competing Interests: The authors have declared that no competing interests exist.

this multistep progression where PanIN models have been induced by chemicals in rodents or by conditional gene replacement in mice [3–5]. There is currently no means of detecting these PanINs before PDAC develops in humans where these lesions are often microscopic and not readily detected by current imaging methods. The identification of protein markers associated with PanINs would be essential for future work on early detection and treatment of PDAC.

CD90 has been reported to be associated with the development of PDAC [6,7]. CD90 is a highly conserved glycoprotein and is a member of the immunoglobulin superfamily. It lacks a transmembrane domain so attaches to the cell membrane by anchoring glycosyl phosphatidyl inositol (GPI). CD90 includes two glycosylation sites in human but three sites in rodents [8]. It is reported that CD90 expression exists differently in various species, but it shows expression on fibroblasts and brain cells in all species [9]. It takes part in adhesion, migration and fibrosis. In recent years, the important roles of CD90 in cancer have gained attention where it may be a candidate marker for cancer stem cells (CSCs) as shown for esophageal cancer and glioma [10–12]. CD90 has been shown to participate in facilitating melanoma cell adhesion to activated endothelium by interaction with the integrin alphavbeta3 [13]. However, whether CD90 presents or co-expresses with other proteins in PanINs has not yet been investigated.

Prior research has shown that CD24 is a potential protein for detection of CSCs [14]. It participates in tumorigenesis and progression through regulating tumor cell proliferation, cell motility and invasion in many cancers such as ovarian cancer, hepatocellular carcinoma, cervical carcinoma and pancreatic cancer [15–17]. Nestl et al used a rat tumor model of pancreatic cancer to identify CD24 mRNA upregulated during metastatic tumor progression [18]. Another report showed CD24 mRNA was upregulated in the pancreatic cancer cell line S2-013 where CD24 gene was considered metastasis-associated [19]. Moreover, CD24 is expressed not only in PanIN lesions but also in intraductal papillary mucinous neoplasm (IPMN), which is another precursor of PDAC [20,21]. CD24 has also been shown to be an important marker for pancreatic cancer stem cells [22].

In the current study, we have investigated the expression patterns of CD90 and CD24 by immunofluoresence staining in PanINs. The results showed that CD90 was mainly expressed in stroma around lesion ducts, but not in acini and islets in PanINs. CD90 showed a higher expression in PanIN III than PanIN III. CD90 however was negative in IPMNs. CD24 was mainly detected in the cytoplasm and membrane of pancreatic ductal epithelium, especially in the apical epithelium of the duct. We found that CD90 protein expression was around the ducts, but that there was little overlap with CD24 expression. A moderate relationship between CD90 or CD24 protein expression with PanIN stages was observed although CD90 was irrelevant to CD24 in PanINs in this study. Together, CD90 or CD24 expression may have a role in the development and progression of PanINs, and their co-expression may be important for detection and treatment of early stage PanINs before the development of PDAC.

Materials and Methods

Human Tissue Specimens

All clinical samples of pancreatic intraepithelial neoplasia (PanIN) and intraductal papillary mucinous neoplasm (IPMN) were from donors provided by the Department of Pathology in the University of Michigan School of Medicine and the normal human pancreatic tissues were purchased from US Biomax Inc (Rockville, MD). Human tissue specimens included normal pancreas (n = 8), PanIN cases (n = 30) and IPMN cases (n = 26). PanIN cases included PanIN III (n = 17), PanIN III (n = 8) and PanIN III (n = 5) and IPMNs comprised low grade IPMN cases (n = 14) and high grade IPMN cases (n = 12). PanINs and IPMNs were diagnosed based

on the pathological sections and clinical symptoms. It should be noted that these slides were from patients that already were diagnosed with PDAC. Tissue specimens obtained by surgical operation were fixed with 4% formaldehyde solution. They were embedded in paraffin after immersing in a series of ethanol/xylene solutions. Formalin-fixed paraffin embedded tissue samples were sliced into 5μm sections. This study was approved by the University of Michigan (IRB00001996).

Hematoxylin and Eosin (HE) Staining

Paraffin sections were stained with hematoxylin and eosin (HE) in order to verify the pathological structure of PanINs and IPMNs. Tissue sections were deparaffinized in xylene for 10 minutes and rehydrated in various concentrations of alcohol (100% ethanol twice, 95% ethanol, 70% ethanol, 3 minutes each). The sections were rinsed in 10% hematoxylin (Sigma-Aldrich, USA) for 3 minutes and decolorized in 0.1% hydrochloric-alcohol solution for 4 seconds. The slides were then counterstained in alcoholic eosin solution (Sigma-Aldrich, USA) for 2 seconds following rinsing in 95% ethanol for 30 seconds. Finally, these sections were dehydrated in a series of alcohol solutions and cover-slipped.

Immunofluoresence Staining

Protein expression was determined by immunofluoresence (IF) staining [7]. Briefly, the tissue specimens were placed in xylene twice for 20 minutes and hydrated in 100% ethanol, 95% ethanol, 75% ethanol, 5 minutes each and water for 3 minutes. Antigen retrieval was completed by boiling the sections in solution (BioGenex HK080-9K) for 15 minutes. Subsequently, these were blocked with 2% BSA in PBS containing 0.05% Tween (PBST) for 1 hour at room temperature and incubated with rabbit anti-human CD90 antibody (1:100, Abcam, Cambridge, MA) or mouse anti-human CD24 antibody (1:150, Abcam, Cambridge, MA) at 4°C overnight. The secondary antibodies of DyLight 488 anti-rabbit IgG (green) or DyLight 549 anti-mouse IgG (red, Vector Laboratories, Burlingame, CA) were used at 1:150 dilution and incubated in a dark place for 1 hour at room temperature. CD90 staining was visualized in green and CD24 in red. Staining with DAPI (blue, 1:4000 dilution) presented nuclei visualization. The sections were dehydrated in alcohol and xylene, then cover-slipped with TM mounting medium (Sigma, USA).

Immunofluoresence Double Staining

IF double staining protocol was used in order to determine the relative expression of CD90 with CD24 and also the expression difference between CD90 and CD45 or α-SMA. Briefly, the sections were placed in antigen retrieval citrate solution heated at 92–98°C for 15 minutes. Then these sections were blocked with 2% BSA at room temperature. The primary antibodies used were anti-human CD90 with anti-human CD24, mouse anti-human α-SMA (1:400, Sigma) or mouse anti-human CD45 (1:100, Abcam, Cambridge, MA), respectively. These antibodies were added to cover sections and were incubated at 4°C overnight. DyLight 488 anti-rabbit IgG (green) and DyLight 549 anti-mouse IgG (red, Vector laboratories, Burlingame, CA) were incubated (1:150 dilution respectively) in a dark place under normal conditions for 1 hour. Nuclei visualization was performed by incubating DAPI (blue) for 15 minutes. These were washed with PBST for 15 minutes between each step. The sections were finally dehydrated and mounted.

Evaluation of Protein Expression

IF slides were scored independently by two investigators (X.P and J.Z). A multiplication product (0–12) was calculated according to the percentage of positive cells (%pos; 0 = no staining, 1 = <10%, 2 = 11%-50%, 3 = 51%-80%, 4>80%) and stain intensity (0 = negative, 1 = weak, 2 = moderate, 3 = strong) for each case. The percentage of positive cells was the mean of percent positive in three different fields under 200× magnification on the fluorescence microscope of a Nikon Eclipse Ti through imaging software NIS-Elements AR. 4.13.00. Every case was divided into a positive- (product>2) or negative-expression (product ≤ 2) group according to the multiplication product for each marker. The positive rate was subsequently calculated in each group.

Statistical Analysis

Protein expression was analyzed using SPSS 16.0 software. Fisher's exact test was applied to analyze the positive rates of multiple groups. One-way ANOVA and Tamhane's test was used to compare the significant differences of protein expression between various groups. Spearman rank correlation was applied to assess the correlation between protein expression and clinicopathological features. A P-value <0.05 was considered as statistically significant in all analyses.

Results

Expression of CD90 in PanIN Lesions

We confirmed firstly the pathological grades of all cases by HE staining. PanIN lesions are divided into three grades depending on the structure and nucleus variation. As shown in Fig 1, PanIN III (low-grade heteromorphosis) was characterized by columnar or papillary epithelial cells as well as minimal cytological atypia. PanIN III (intermediate-grade) presents the atypical changes of the nucleus where polarity disappears, and also nuclear enlargement and pseudostratified epithelium occurs. The important features of PanIN III (high-grade) include nuclear atypia hyperplasia, commonly the papillary or micro papillary morphology and the dystrophic goblet cell, which belongs to noninvasive lesions. Moreover, part of the epithelial cells of damaged ducts is missing in PanIN III.

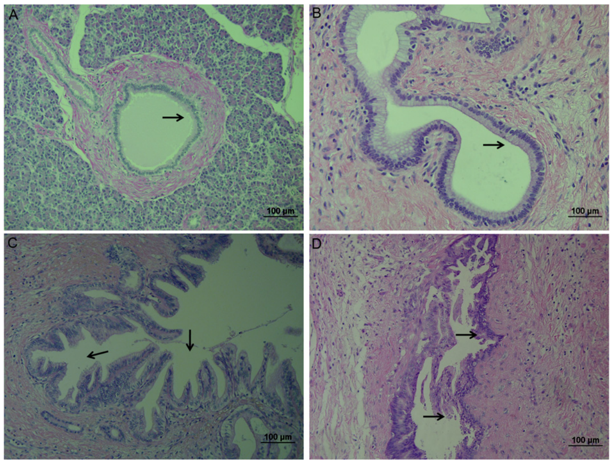

Fig 1. Three PanIN hispathological grades (HE). A:normal pancreas; B:PanIN III, minimal cytological atypia (arrow); C:PanIN III, nuclear polarity disappears, moderate cytological atypia (arrow); D: PanIN III, nuclear atypia hyperplasia, papillary or micro papillary morphology (arrow). Scale bars = 100 μm.

doi:10.1371/journal.pone.0158021.g001

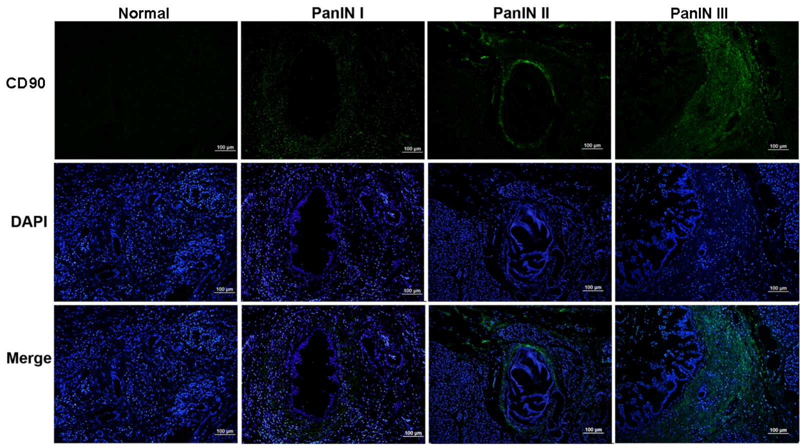

Fig 2. Expression of CD90 (green) in normal and various PanIN tissues. Nuclei visualization was shown by DAPI (blue) staining. There was no CD90 expression in the ducts or acini in normal tissue. CD90 expression was mainly seen in stromal cells around lesion ducts, and there was no expression observed in acini of PanIN tissues. CD90 expression increased gradually with PanIN grades. Scale bars = 100 μm.

doi:10.1371/journal.pone.0158021.g002

CD90 expression in PanINs was then evaluated by IF staining. Our previous tests have verified that the rabbit anti-human CD90 antibody has the specificity which is suitable for the immunostaining test [7]. In normal tissue, there was no CD90 expression in the ducts or acini of the exocrine glands as well as the islets of the endocrine gland, but there was weak expression in connective tissue (Fig 2). The negative rate of CD90 expression was 8 (100%) of 8 according to this negative standard (multiplication product ≤ 2, Table 1), and the CD90 staining mean was 0.375, which indicates that CD90 had weak and sparse expression in normal pancreas.

As shown in Fig 2, CD90 expression in PanIN lesions was mainly observed in stroma around the lesion duct, and there was no expression observed in acini and islets. Twenty three cases (77%) of PanINs were positive, which included 11 (65%) of 17 in PanIN III, 7 (87%) of 8 in PanIN III and 5 (100%) of 5 in PanIN III (Table 1). The results showed the positive rates in PanIN III, III and III did significantly differ from normal pancreas at $P<0.01$ level (Table 1) by Fisher's exact test. We estimated the CD90 differential expression and the connection with pathological grade according to stained area and intensity. One-way ANOVA was used to indicate there was a significant difference [$F_{(3,34)} = 12.04, P = 0.00$] in normal and PanIN groups. The statistical results of Tamhane's test indicated that the mean scores in three PanIN groups ($M_{III} = 4.65$, $M_{III} = 6.00$, $M_{III} = 10.00$) were higher than in normal pancreas, respectively ($P_{III} = 0.0006$, $P_{III} = 0.01$, $P_{III} = 0.00$). Also, CD90 showed stronger expression in PanIN III than in PanIN III ($P = 0.003$, Fig 3A). CD90 expression thus increases gradually with disease development. The Spearman's correlation coefficient (R) is 0.562 ($P = 0.0002$) in all PanINs, which

Table 1. Relationship between CD90, CD24 expression and the histologic grade of PanIN.

Histologic grade	CD90 expression			CD24 expression		
	Negative	Positive	*P**	Negative	Positive	*P**
Normal pancreas	8 (100%)	0 (0%)		8 (100%)	0 (0%)	
PanIN I	6 (35%)	11 (65%)	0.003	7 (41%)	10 (59%)	0.018
PanIN II	1 (13%)	7 (87%)	0.001	1 (13%)	7 (87%)	0.001
PanIN III	0 (0%)	5 (100%)	0.001	0 (0%)	5 (100%)	0.001

* *PanIN VS* corresponding normal pancreas

doi:10.1371/journal.pone.0158021.t001

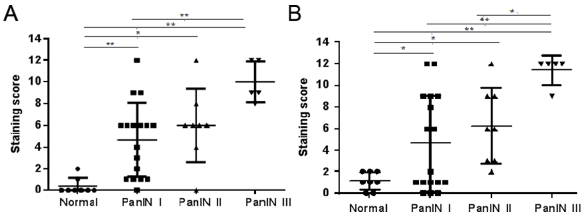

Fig 3. Comparison of staining score of CD90 and CD24 expression between PanIN and normal pancreas. A: CD90 expression; B: CD24 expression. *means $P<0.05$;**means $P<0.01$.

doi:10.1371/journal.pone.0158021.g003

indicated there was moderate correlation between CD90 expression and hispathological grade. These results taken together suggest that for CD90 there is significant association of its expression with the pathological grade of PanIN.

Expression of CD24 in PanINs Associated with the Histologic Grade

In normal pancreas, there was rare or negative expression in acinar cells, where no CD24 expression was observed in pancreatic ductal epithelium, islets and stroma cells (Fig 4). The results from PanINs showed that CD24 was mainly present in the cytoplasm and membrane of pancreatic ductal epithelium, especially in the apical epithelium of the duct (Fig 4). It is clear that the ductal epithelium was stained with red indicating the presence of CD24 except for the nucleus which stained blue for DAPI. Its expression appeared in all PanINs, and there was strong expression in all PanIN III cases (Fig 4).

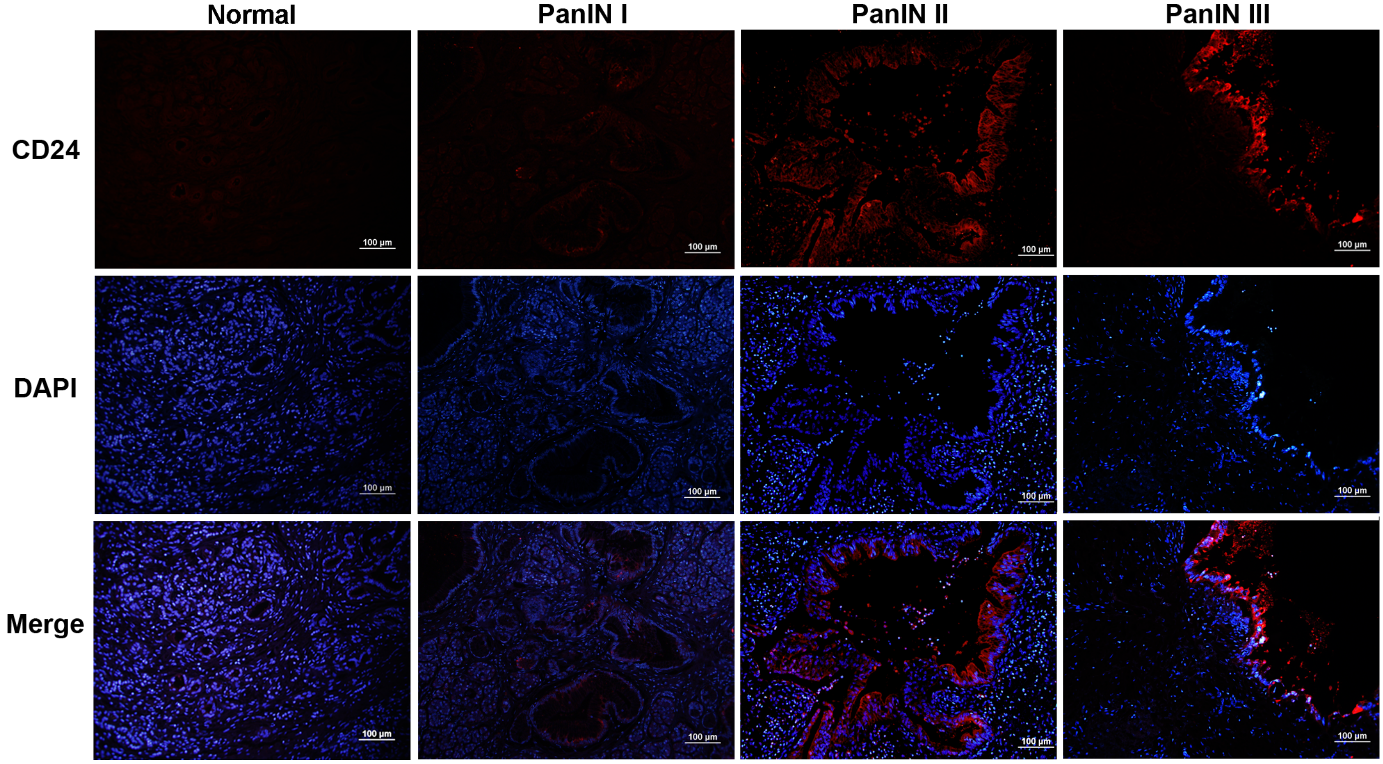

Fig 4. Expression of CD24 (red) in normal and various PanIN tissues. Nuclei visualization was shown by staining DAPI (blue). CD24 presented weak or negative expression in acinar cells, and no staining was observed in pancreatic ductal epithelium in the normal goup. CD24 was mainly present in the cytoplasm and membrane of the pancreatic ductal epithelium, especially in the apical epithelium of the duct (arrow). Scale bars = 100 μm.

doi:10.1371/journal.pone.0158021.g004

To evaluate the difference and relationship between CD24 expression and pathological grade, we assessed the staining scores in all cases of PanIN. The positive rates of CD24 expression in normal pancreas, PanIN III, III and III were 0(0%) of 8, 10(59%) of 17, 7(87%) of 8 and 5 (100%) of 5 (Table 1). The statistical results presented here were the significant differences of the positive rate in three stages of PanIN compared with the normal group, respectively (all $P<0.05$, Table 1). The results of Tamhane's test showed not only that all PanIN groups existed but there was an increase in staining scores compared with normal pancreas, respectively ($P_{III} = 0.037$, $P_{III} = 0.025$, $P_{III} = 0.00$), where CD24 had stronger expression in PanIN III ($M_{III} = 11.4$) compared with PanIN IIIIII ($M_{III} = 6.25$, $P = 0.00$) or PanIN III ($M_{III} = 4.58$, $P = 0.025$). Correlation analysis showed there was a moderate relationship between CD24 expression and PanIN stages ($R = 0.638$, $P = 0.00$). Therefore, CD24 is an important marker for PanINs.

Co-expression of CD90 with CD24

Double IF was used to examine the co-expression of CD90 with CD24 in all cases. This double staining was relevant to the damaged ducts, where CD24 (red) was observed in the cytoplasm and membrane of ductal epithelium and CD90 expression (green) was observed in stromal cells around the ducts (Fig 5). The expression of these two proteins showed little overlap in PanIN tissues. We evaluated the relationship between CD90 and CD24 expression differences in PanIN grades in terms of the increasing trend of whether CD90 or CD24 expression was

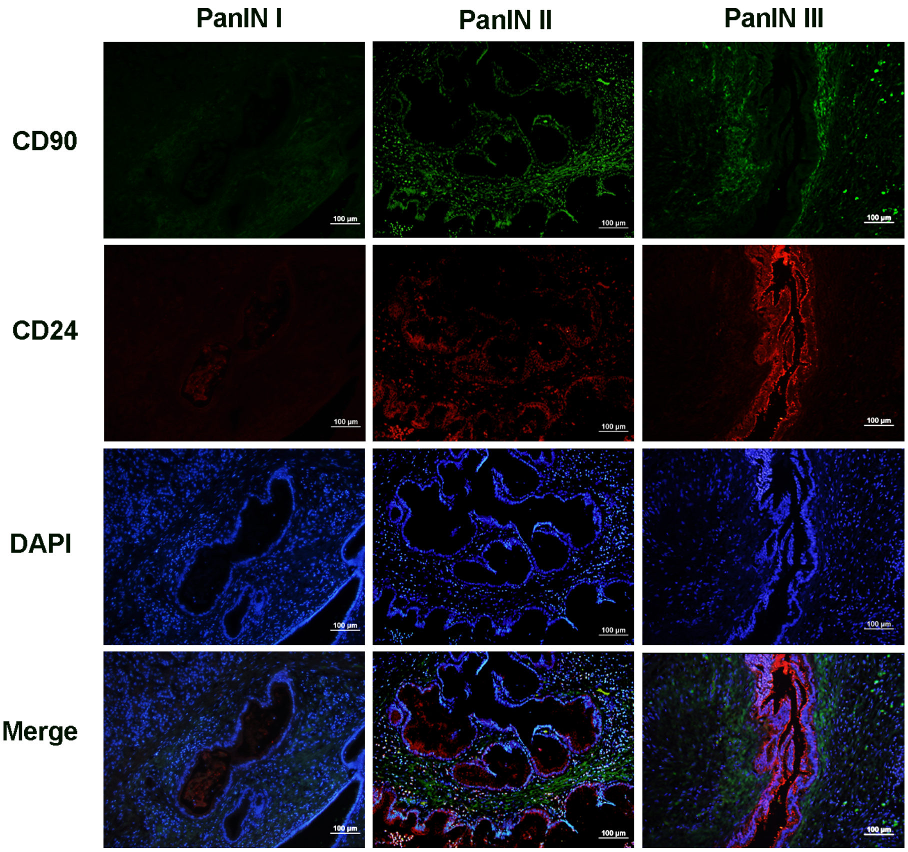

Fig 5. Co-expression of CD90 (green) and CD24(red) in PanIN. Nuclei visualization was shown by staining DAPI (blue). CD90 mainly presented in stromal cells, however, CD24 expressed in cytoplasm and membrane of pancreatic ductal epithelium. There was little overlap in all stages. Scale bars = 100 μm.

doi:10.1371/journal.pone.0158021.g005

seen based on the mutiplication products. The results showed there was no correlation of CD24 and CD90 expression in three PanIN groups (R = 0.284, P = 0.128) although there was a significant relationship between their expression (R = 0.426, P = 0.008) when PanIN and normal tissues were all counted. This indicates that CD90 expression in PanINs is not linked to CD24 expression in this study.

Double Staining of CD90 with α-SMA

Pancreatic stellate cells (PSCs) marked by α-smooth muscle actin (α-SMA) expression exist in pancreatic stromal cells which are closely related to fibroblastic proliferation and fibrosis in PDAC. Our previous work had shown that CD90 mostly overlaps with α-SMA in stroma of PDAC [7]. Here we further investigated the relationship between CD90 and α-SMA in PanIN III by double IF. The result showed that α-SMA presented in the activated PSCs surrounding the damaged ducts. There was strong overlap between the expression of CD90 and α-SMA in PanIN III (Fig 6), which suggests activated PSCs also express CD90 protein.

Co-expression of CD90 with CD45

Human CD90 regulates inflammatory cell recruitment induced by immune response [23]. It is reported that there is CD90 expression on a subpopulation of leukocytes in HIV-infected patients [24]. To eliminate CD90 expression from leukocytes, we stained for the double expression of CD90 and CD45, which is leukocyte common antigen (LCA) and is expressed in all leukocytes. There was negative CD45 expression in PanINs (Fig 7), which indicated that CD90 expression in PanINs was not from leukocytes.

Discussion

Based on prior work there are several proteins including CD133, CD90 and CD24 which may be good markers of PanINs. CD133 is a marker for many stem cells which has been used to identify putative CSC from solid tumors [25–27]. Kazuya S et al reported that CD133 staining was not observed in all stages of PanIN or IPMN and only expressed in PDAC, which indicates that it can differentiate IPMN from PDAC but not PanIN from IPMN [28]. CD24 which is involved in cell adhesion and tumor metastasis is expressed not only in PanINs and IPMNs but also in PDAC, which means that like CD133 it does not distinguish between PanINs and IPMNs. Our previous studies have confirmed that CD90 has a different expression pattern between various cancers. CD90 expresses on the liver tumor islands or parenchymal, instead of the stroma, and there is co-expression of CD90 with CD24 and CD133 just on $CD90^+$ liver cancer cells and not on other cells [29]. However, in PDAC there is over-expression of CD90

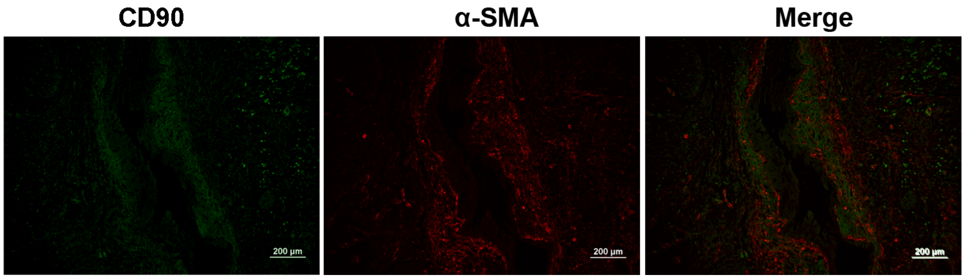

Fig 6. Co-expression of CD90 (green) and α-SMA (red) in PanIN III. α-SMA was present in the activated PSCs surrouding the damaged ducts. There was strong overlap between the expression of CD90 and α-SMA. Scale bars = 200 µm.

doi:10.1371/journal.pone.0158021.g006

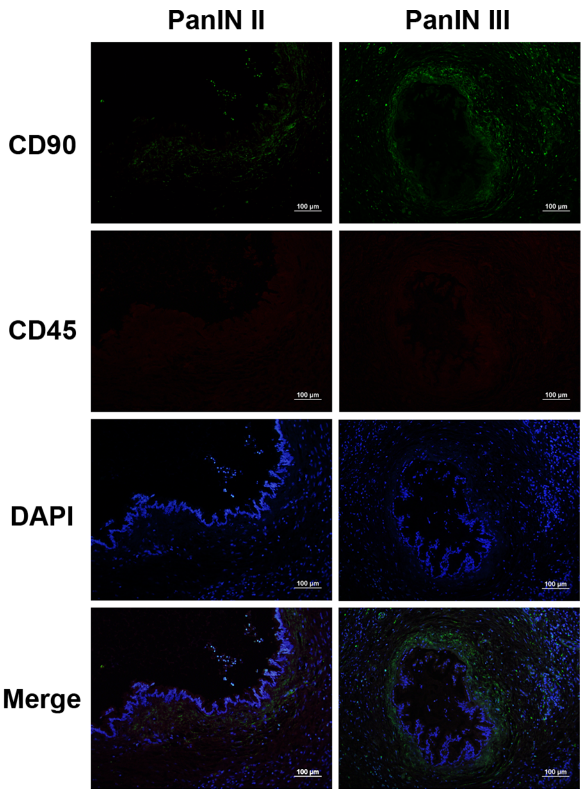

Fig 7. Representative images of CD90 (green) with CD45(red) both in PanIN III and PanIN III. Nuclei visualization was shown by staining DAPI (blue). CD45 was negative in PanINs. Scale bars = 100 μm.

doi:10.1371/journal.pone.0158021.g007

mainly in the stroma which is connected with tumor growth in all stages and CD90 is expressed together with CD24 but on different cells [7,30]. CD90 is regarded as a marker of several types of CSC where reports on CD90 expression in PanINs are still limited. We thus explored PanINs in terms of CD90 expression and then co-expression with CD24 in this study.

We studied CD90 expression in normal and PanINs tissues after the pathological grade had been confirmed by HE staining. CD90 presented positively in three PanIN stages and showed gradually enhanced expression with various grades of PanIN, where the interference of CD45 expression from leukocytes was ruled out. CD90 had more significant expression in PanIN III than in PanIN III (Fig 3A) in this study. Our previous study on PDAC indicated CD90 presented strongly in stromal cells such as PSCs, and CD90 was significantly relevant to PDAC through statistical analysis [7]. Together, CD90 was expressed during the progression from PanIN III to PDAC, which may be related to a promoting role in the development of PanIN or PDAC. In comparison, there was negative CD90 expression observed in stroma around lesion ducts both in low and high grades in IPMN tissue which also developed into PDAC (S1 Fig). Therefore, CD90 may be a potential marker to distinguish PanINs from IPMNs.

The microenvironment of diseases is composed of many components, such as stromal cells, extracellular matrix and many cytokines, which play an important role in the generation and development of diseases, including invasion and metastasis of tumor cells [31,32]. The stromal cells of PDAC include PSCs marked by α-SMA expression. In this study, we found that α-SMA protein was expressed in activated PSCs around the lesion ducts of PanIN III, which is consistent in the expression of α-SMA presented in the area surrounding exclusively ductal lesions in a mouse model [33]. Our work also revealed that activated PSCs expressed CD90 protein. PSCs

contribute to the proliferation and apoptosis of pancreatic cancer cells by secreting type I collagen [34]. CD90 is an important regulator of cell-cell and cell-matrix interactions and is involved in signal transduction and cytokine synthesis. It has been reported that anti-CD90 mAb inhibited cell proliferation and affected apoptosis in T-cell and B-cell lymphoma cells, which mean anti-CD90 mAb has an antiproliferative effect in vitro [35,36]. CD90 may possibly affect the progress of PanIN through regulating the microenvironment. However, its specific role in the tumor is still controversial where CD90 shows inhibitory action in some tumours such as neuroblastoma and ovarian cancer [37,38].

CD24 expression was found to increase at an early stage of PanIN. Our results indicate that the staining score was significantly stronger in all stages of PanIN compared to normal pancreas and increased CD24 expression was related to the degree of PanIN lesions (Fig 3B). However, others have reported significantly increased CD24 expression was found only in PanIN III compared with normal tissue [20]. We now observe different results where CD24 stained in PanIN III and III. There may be several reasons for these differences. In these other studies, the percentage of positive cells (0–100%) was chosen as the evaluated index of marker expression. In our study we used the multiplication product of the percentage of positive cells and stain intensity as an evaluation indicator, which may reflect the protein expression more comprehensively. Also the protein detection method was different where they used an immunohistochemistry (IHC) method in their study, whereas we employed IF staining. The difference of PanIN cases selected might be one of the reasons.

There are many functions for CD24 such as cell-cell interaction and metastasis, however other functions are still unknown. CD24 is a membrane receptor anchored by glycosyl-phosphotidylinositol. Many reports have shown that CD24 expresses not only in the membrane but also in the cytoplasm in PDAC and colorectal cancer [7,39]. An intracellular study on pancreatic cancer indicated CD24 expression both in membrane and cytoplasm impeded cell invasion and metastasis. Our previous studies though showed that $CD24^+CD44^+$ cells isolated from a human pancreatic carcinoma cell line (PANC-1) had 4-fold increased invasion ability compared to $CD24^-CD44^+$ cells [40]. PanIN is a noninvasive ductal lesion, which does not pass through the basement membrane. The results in this study showed that CD24 was found on and in pancreatic ductal epithelium and there was moderate correlation between CD24 expression and the histologic grade of PanIN (Figs 3B and 4). CD24 was involved not only in all stages of PanIN but also in the progression from PanIN to PDAC. This suggests that CD24 expression in PanINs might be related to the progression from noninvasive to an invasive lesion.

A single marker does not represent the disease even though many biomarkers are probably involved in the progression of PDAC [41,42]. The stromal cells in PDAC obstruct the discovery of disease as damaged epithelial cells are in the minority [43]. Exploring the co-expression of markers in epithelial and stromal cells is regarded as a potential method to detect the disease. We further studied as to whether CD90 and CD24 proteins have the same co-expression in PanINs as in PDAC [7]. CD24 was observed here in the pancreatic epithelium in the lesion ducts, surrounded by stroma expressing CD90 protein outside the lesion ducts in all PanIN grades (Fig 5), and there was no obvious relationship between CD90 and CD24 expression. The difference of expression location and pattern may determine the conditions under which CD90 and CD24 play various roles in PanINs. The co-expression of these two proteins may be essential for detection in the early stages of PanIN, which will be important for diagnosis and treatment of PDAC.

Conclusion

We have found that CD90 expression is a potential marker to distinguish PanINs from IPMNs. We also found that increased CD90 and CD24 expression presented at an early stage and are related to the development of PanIN lesions. Their co-expression might play an important role in early detection of PanINs. This may be useful where many of these lesions are microscopic and may be difficult for a pathologist to find on a tissue section. There are also cases of ducts which have a mix of normal and PanINs on the same ducts. The use of these staining markers would enhance detection of these small lesions.

Supporting Information

S1 Fig. Expression of CD90 in IPMN. Nuclei visualization was shown by DAPI (blue) staining. There was no CD90 expression observed in stroma around the lesion ducts both in low and high grades of IPMN. Scale bars = 100 μm.
(TIF)

Acknowledgments

We thank Dr. Min Zhang (University of Michigan) for her constructive suggestions on the statistical analysis in this study.

Author Contributions

Conceived and designed the experiments: DML JZ. Performed the experiments: XP JZ RY JS ZT. Analyzed the data: XP MA. Contributed reagents/materials/analysis tools: JZ JS ZT. Wrote the paper: XP DML.

References

1. Hruban RH, Takaori K, Klimstra DS, Adsay NV, Albores-Saavedra J, Biankin AV, et al. An illustrated consensus on the classification of pancreatic intraepithelial neoplasia and intraductal papillary mucinous neoplasms. Am J Surg Pathol. 2004; 28 (8):977–87. PMID: 15252303
2. Hernandez YG, Lucas AL. MicroRNA in pancreatic ductal adenocarcinoma and its precursor lesions. World J Gastrointest Oncol. 2016; 8(1):18–29. doi: 10.4251/wjgo.v8.i1.18 PMID: 26798434
3. Distler M, Aust D, Weitz J, Pilarsky C, Grutzmann R. Precursor lesions for sporadic pancreatic cancer: PanIN, IPMN, and MCN. BioMed Res Int. 2014; doi: 10.1155/2014/474905
4. Wang L, Liu HL, Li Y, Yuan P. Proteomic analysis of pancreatic intraepithelial neoplasia and pancreatic carcinoma in rat models. World J Gastroenterol. 2011; 17 (11):1434–41. doi: 10.3748/wjg.v17.i11.1434 PMID: 21472101
5. Ying H, Kimmelman AC, Lyssiotis CA, Hua S. Chu GC, Fletcher-Sananikone E, et al. Oncogenic Kras maintains pancreatic tumors through regulation of anabolic glucose metabolism. Cell. 2012; 149(3): 656–70. doi: 10.1016/j.cell.2012.01.058 PMID: 22541435
6. Foygel K, Wang H, Machtaler S, Lutz AM, Chen R, Pysz M, et al. Detection of pancreatic ductal adenocarcinoma in mice by ultrasound imaging of thymocyte differentiation antigen 1. Gastroenterology. 2013; 145(4):7885–94.
7. Zhu J, Thakolwiboon S, Liu X, Zhang M, Lubman DM. Overexpression of CD90 (Thy-1) in pancreatic adenocarcinoma present in the tumor microenvironment. PLoS One. 2014; 9(12):e115507. doi: 10.1371/journal.pone.0115507 PMID: 25536077
8. Loertscher R, Lavery P. The role of glycosyl phosphatidyl inositol (GPI)-anchored cell surface proteins in T-cell activation. Transpl Immunol. 2002; 9(2–4):93–6. PMID: 12180852
9. Pont S. Thy-1: a lymphoid cell subset marker capable of delivering an activation signal to mouse T lymphocytes. Biochimie. 1987; 69 (4):315–20. PMID: 2888493
10. He J, Liu Y, Zhu T, Zhu J, Dimeco F, Vescovi AL, et al. CD90 is identified as a candidate marker for cancer stem cells in primary high-grade gliomas using tissue microarrays. Mol Cell Proteomics. 2012; 11 (6):M111.010744.

11. Tang KH, Dai YD, Tong M, Chan YP, Kwan PS, Fu L, et al. A CD90(+) tumor-initiating cell population with an aggressive signature and metastatic capacity in esophageal cancer. Cancer Res. 2013; 73 (3):2322–32.
12. He J, Zhu J, Liu Y, Wu J, Nie S, Heth JA, et al. Immunohistochemical staining, laser capture microdissection, and filter-aided sample preparation-assisted proteomic analysis of target cell populations within tissue samples. Electrophoresis. 2013; 34(11):1627–1636. doi: 10.1002/elps.201200566 PMID: 23436586
13. Saalbach A, Wetzel A1, Haustein UF, Sticherling M, Simon JC, Anderegg U. Interaction of human Thy-1 (CD90) with the integrin alphavbeta3 (CD51/CD61): an important mechanism mediating melanoma cell adhesion to activated endothelium. Oncogene. 2005; 24(29):4710–20. PMID: 15897908
14. Chen Y, Song J, Jiang Y, Yu C, Ma Z. Predictive value of CD44 and CD24 for prognosis and chemotherapy response in invasive breast ductal carcinoma. Int J Clin Exp Pathol. 2015; 8(9):11287–95. PMID: 26617852
15. Zhao J, Xu G, Qian YW, Li YW. Down-regulation of miR-205 promotes stemness of hepatocellular carcinoma cells by targeting PLCβ1 and increasing CD24 expression. Neoplasma. 2015; 62(4):567–73. doi: 10.4149/neo_2015_068 PMID: 25997960
16. Kwon GY, Ha H, Ahn G, Park SY, Huh SJ, Park W, et al. Role of CD24 protein in predicting metastatic potential of uterine cervical squamous cell carcinoma in patients treated with radiotherapy. Int J Radiat Oncol Biol Phys. 2007; 69(4):1150–6. PMID: 17692475
17. Shankar S, Nall D, Tang SN, Meeker D, Passarini J, Sharma J, et al. Resveratrol inhibits pancreatic cancer stem cell characteristics in human and KrasG12D transgenic mice by inhibiting pluripotency maintaining factors and epithelial-mesenchymal transition. PLoS One. 2011; 6(1):e16530. doi: 10.1371/journal.pone.0016530 PMID: 21304978
18. Nestl A1, Von Stein OD, Zatloukal K, Thies WG, Herrlich P, Hofmann M, et al. Gene expression patterns associated with the metastatic phenotype in rodent and human tumors. Cancer Res. 2001; 61 (4):1569–77. PMID: 11245467
19. Kohlgraf KG1, Gawron AJ, Higashi M, Meza JL, Burdick MD, Kitajima S, et al. Contribution of the MUC1 tandem repeat and cytoplasmic tail to invasive and metastatic properties of a pancreatic cancer cell line. Cancer Res. 2003 15; 63(16):5011–20. PMID: 12941828
20. Kure S, Matsuda Y, Hagio M, Ueda J, Naito Z, Ishiwata T, et al. Expression of cancer stem cell markers in pancreatic intraepithelial neoplasias and pancreatic ductal adenocarcinomas. Int J Oncol. 2012; 41 (4):1314–24. doi: 10.3892/ijo.2012.1565 PMID: 22824809
21. Ikenaga N, Ohuchida K, Mizumoto K, Yu J, Kayashima T, Hayashi A, et al. Characterization of CD24 expression in intraductal papillary mucinous neoplasms and ductal carcinoma of the pancreas. Hum Pathol. 2010; 41(10):1466–74. doi: 10.1016/j.humpath.2010.04.004 PMID: 20619441
22. Jacob J, Bellach J, Grutzmann R, Alldinger I, Pilarsky C, Dietel M, et al. Expression of CD24 in adenocarcinomas of the pancreas correlates with higher tumor grades. Pancreatology. 2004; 4(5):454–60. PMID: 15256807
23. Haeryfar SM, Hoskin DW. Thy-1: more than a mouse pan-T cell marker. J Immunol. 2004; 173 (6):3581–8. PMID: 15356100
24. Guillot-Delost M, Le Gouvello S, Mesel-Lemoine M, Cherai M, Baillou C, Simon A, et al. Human CD90 identifies Th17/Tc17 T cell subsets that are depleted in HIV-infected patients. J Immunol. 2012; 188 (3):981–91. doi: 10.4049/jimmunol.1101592 PMID: 22184726
25. Triviai I, Stubig T, Niebuhr B, Hussein K, Tsiftsoglou A, Fehse B, et al. CD133 marks a stem cell population that drives human primary myelofibrosis. Haematologica. 2015; 100(6):768–79. doi: 10.3324/haematol.2014.118463 PMID: 25724578
26. Bi Y, Meng Y, Wu H, Cui Q, Luo Y, Xue X, et al. Expression of the potential cancer stem cell markers CD133 and CD44 in medullary thyroid carcinoma: A ten-year follow-up and prognostic analysis. J Surg Oncol. 2016; 113(2):144–51. doi: 10.1002/jso.24124 PMID: 26799258
27. Rasheed ZA, Matsui W. Biological and clinical relevance of stem cells in pancreatic adenocarcinoma. J Gastroenterol Hepatol. 2012; Suppl 2: :15–8. doi: 10.1111/j.1440-1746.2011.07015.x PMID: 22320910
28. Shimizu K, Itoh T, Shimizu M, Ku Y, Hori Y. CD133 expression pattern distinguishes intraductal papillary mucinous neoplasms from ductal adenocarcinomas of the pancreas. Pancreas. 2009; 38(8): e207–214. doi: 10.1097/MPA.0b013e3181bb5037 PMID: 19786935
29. Thakolwiboon S, Zhu J, Liang Q, Welling TH, Zhang M, Lubman DM, et al. Heterogeneity of the CD90 population in different stages of hepatocarcinogenesis. J Proteomics Bioinform. 2014; 7(10):296–302. PMID: 25580065

30. Zhu J, Nie S, Wu J, Lubman DM. Target proteomic profiling of frozen pancreatic $CD24^+$ adenocarcinoma tissues by immuno-laser capture microdissection and nano-LC-MS/MS. J Proteome Res. 2013; 12(6):2791–804. doi: 10.1021/pr400139c PMID: 23679566

31. Fujita Y, Yoshioka Y, Ochiya T. Extracellular vesicle transfer of cancer pathogenic components. Cancer Sci. 2016; doi: 10.1111/cas.12896

32. Codony-Servat J, Rosell R. Cancer stem cells and immunoresistance: clinical implications and solutions. Transl Lung Cancer Rse. 2015; 4(6):689–703.

33. Garcia-Carracedo D, Yu CC, Akhavan N, Fine SA, Schönleben F, Maehara N, et al. Smad4 loss synergizes with TGFα overexpression in promoting pancreatic metaplasia, PanIN development, and fibrosis. PLoS One. 2015; 10(3):e0120851. doi: 10.1371/journal.pone.0120851 PMID: 25803032

34. Armstrong T, Packham G, Murphy LB, Bateman AC, Conti JA, Fine DR, et al. Type I collagen promotes the malignant phenotype of pancreatic ductal adenocarcinoma. Clin Cancer Res. 2004; 10(21):7427–37. PMID: 15534120

35. Ishiura Y1, Kotani N, Yamashita R, Yamamoto H, Kozutsumi Y, Honke K. Anomalous expression of Thy1 (CD90) in B-cell lymphoma cells and proliferation inhibition by anti-Thy1 antibody treatment. Biochem Biophys Res Commun. 2010; 396(2):329–34. doi: 10.1016/j.bbrc.2010.04.092 PMID: 20403334

36. Fujita N1, Kato Y, Naito M, Tsuruo T. A novel anti-Thy-1 (CD90) monoclonal antibody induces apoptosis in mouse malignant T-lymphoma cells in spite of inducing bcl-2 expression. Int J Cancer. 1996 May 16; 66(4):544–50. PMID: 8635872

37. Lehmann GM, Woeller CF, Pollock SJ, O'Loughlin CW, Gupta S, Feldon SE, et al. Novel anti-adipogenic activity produced by human fibroblasts. Am J Physiol Cell Physiol. 2010; 299(3):C672–81. doi: 10.1152/ajpcell.00451.2009 PMID: 20554910

38. Bukovsky A, Caudle MR, Carson RJ, Gaytan F, Huleihel M, Kruse A, et al. Immune physiology in tissue regeneration and aging, tumor growth, and regenerative medicine. Aging (Albany NY). 2009; 1(2): 157–81.

39. Cherciu I, Bărbălan A, Pirici D, Mărgăritescu C, Săftoiu A. Stem cells, colorectal cancer and cancer stem cell markers correlations. Curr Health Sci J. 2014; 40(3):153–61. doi: 10.12865/CHSJ.40.03.01 PMID: 25729599

40. Zhu J, He J, Liu Y, Simeone DM, Lubman DM. Identification of glycoprotein markers for pancreatic cancer CD24+CD44+ stem-like cells using nano-LC-MS/MS and tissue microarray. J Proteome Res. 2012; 11(4):2272–81. doi: 10.1021/pr201059g PMID: 22335271

41. Kwon MS, Kim Y, Lee S, Namkung J, Yun T, Yi SG, et al. Integrative analysis of multi-omics data for identifying multi-markers for diagnosing pancreatic cancer. BMC Genomics. 2015; 16 Suppl 9:S4. doi: 10.1186/1471-2164-16-S9-S4 PMID: 26328610

42. Cid-Arregui A, Juarez V. Perspectives in the treatment of pancreatic adenocarcinoma. World J Gastroenterol. 2015; 21(31):9297–316. doi: 10.3748/wjg.v21.i31.9297 PMID: 26309356

43. Moffitt RA, Marayati R, Flate EL, Volmar KE, Loeza SG, Hoadley KA, et al. Virtual microdissection identifies distinct tumor- and stroma-specific subtypes of pancreatic ductal adenocarcinoma. Nat Genet. 2015; 47(10):1168–78. doi: 10.1038/ng.3398 PMID: 26343385
